# Comparison of next generation sequencing technologies for transcriptome characterization

**DOI:** 10.1186/1471-2164-10-347

**Published:** 2009-08-01

**Authors:** P Kerr Wall, Jim Leebens-Mack, André S Chanderbali, Abdelali Barakat, Erik Wolcott, Haiying Liang, Lena Landherr, Lynn P Tomsho, Yi Hu, John E Carlson, Hong Ma, Stephan C Schuster, Douglas E Soltis, Pamela S Soltis, Naomi Altman, Claude W dePamphilis

**Affiliations:** 1 Department of Biology, Institute of Molecular Evolutionary Genetics, and The Huck Institutes of the Life Sciences, The Pennsylvania State University, University Park, PA 16802, USA; 2 Department of Plant Biology, University of Georgia, Athens, GA 30602, USA; 3 Department of Biology, University of Florida, PO Box 118526, Gainesville, FL, 32611, USA; 4 The School of Forest Resources, Department of Horticulture, and Huck Institutes of the Life Sciences, Pennsylvania State University, 323 Forest Resources Building, University Park, PA 16802, USA; 5 Center for Comparative Genomics, Center for Infectious Disease Dynamics, The Pennsylvania State University, University Park, PA 16802, USA; 6 Florida Museum of Natural History, University of Florida, P.O. Box 117800, Gainesville, FL, 32611, USA; 7 Department of Statistics and The Huck Institutes of the Life Sciences, The Pennsylvania State University, University Park, PA 16802, USA

## Abstract

**Background:**

We have developed a simulation approach to help determine the optimal mixture of sequencing methods for most complete and cost effective transcriptome sequencing. We compared simulation results for traditional capillary sequencing with "Next Generation" (NG) ultra high-throughput technologies. The simulation model was parameterized using mappings of 130,000 cDNA sequence reads to the *Arabidopsis *genome (NCBI Accession SRA008180.19). We also generated 454-GS20 sequences and *de novo *assemblies for the basal eudicot California poppy (*Eschscholzia californica*) and the magnoliid avocado (*Persea americana*) using a variety of methods for cDNA synthesis.

**Results:**

The *Arabidopsis *reads tagged more than 15,000 genes, including new splice variants and extended UTR regions. Of the total 134,791 reads (13.8 MB), 119,518 (88.7%) mapped exactly to known exons, while 1,117 (0.8%) mapped to introns, 11,524 (8.6%) spanned annotated intron/exon boundaries, and 3,066 (2.3%) extended beyond the end of annotated UTRs. Sequence-based inference of relative gene expression levels correlated significantly with microarray data. As expected, NG sequencing of normalized libraries tagged more genes than non-normalized libraries, although non-normalized libraries yielded more full-length cDNA sequences. The *Arabidopsis *data were used to simulate additional rounds of NG and traditional EST sequencing, and various combinations of each. Our simulations suggest a combination of FLX and Solexa sequencing for optimal transcriptome coverage at modest cost. We have also developed ESTcalc http://fgp.huck.psu.edu/NG_Sims/ngsim.pl, an online webtool, which allows users to explore the results of this study by specifying individualized costs and sequencing characteristics.

**Conclusion:**

NG sequencing technologies are a highly flexible set of platforms that can be scaled to suit different project goals. In terms of sequence coverage alone, the NG sequencing is a dramatic advance over capillary-based sequencing, but NG sequencing also presents significant challenges in assembly and sequence accuracy due to short read lengths, method-specific sequencing errors, and the absence of physical clones. These problems may be overcome by hybrid sequencing strategies using a mixture of sequencing methodologies, by new assemblers, and by sequencing more deeply. Sequencing and microarray outcomes from multiple experiments suggest that our simulator will be useful for guiding NG transcriptome sequencing projects in a wide range of organisms.

## Background

Sequencing technology has made great advances over the last 30 years since the development of chain-terminating inhibitor-based technologies [1]. Traditional sequencing approaches require cloning of DNA fragments into bacterial vectors for amplification and sequencing of individual templates using vector-based primers. This approach was adapted for cDNA libraries [2] and, with the advent of capillary sequencing, became suitable for high-throughput sequencing of large samples of transcripts, termed Expressed Sequence Tags (ESTs). ESTs have become an invaluable resource for gene discovery, genome annotation, alternative splicing, SNP discovery, molecular markers for population analysis, and expression analysis in animal, plant, and microbial species [3]. Other approaches for analyzing transcriptomes include serial analysis of gene expression (SAGE) [4], massively parallel signature sequencing (MPSS) [5], and microarrays [6,7]. These approaches, which involve the sequencing or hybridizing of small concatamers of cDNA derived from mRNA by reverse transcription, have been used successfully in analyzing the expression of genomes (transcriptomes) at a very large scale, usually from species with a sequenced genome or an existing and extensive EST data set. Although several alternatives have been described since the emergence of EST sequencing projects, none has yet totally supplanted the use of bacterial vectors and Sanger sequencing.

In 2005, two new sequencing technologies were introduced both based on sequencing by synthesis, which promised to replace or enhance traditional sequencing methods. The 454 system http://www.454.com, using pyrosequencing technology [8], and the Solexa system http://www.illumina.com, which detects fluorescence signals [9]. Both execute millions of sequencing reactions in parallel, producing data at ultrahigh rates [10]. Although read lengths are much shorter with these new methods than with capillary sequencing (averaging 100–230 bp and 300–400 bp for 454FLX and 454Titanium, respectively, and 35 to up to 76 b for Illumina Solexa platforms), respectively, both platforms generate sufficient data to completely re-sequence bacterial genomes in a single run [8,11-13]. In the past year, Applied Biosystems has introduced their SOLiD sequencer http://www3.appliedbiosystems.com, another short-read 20–35 bp platform, with read lengths anticipated to be 50 bp in the upcoming SOLiD3 release. The three platforms offer a variety of experimental approaches for characterizing a transcriptome, including single-end and paired-end cDNA sequencing, tag profiling (3' end sequencing especially appropriate to estimating expression level), methylation assays, small RNA sequencing, sample tagging ("barcoding") to permit small subsample identification, and splice variant analyses. Several challenges face investigators hoping to use these methods, including the relatively large cost of most NG experiments and intense demands for data storage and analysis on the scale required for NG datasets, and rapidly evolving technologies. Initial studies reported success with 454 sequencing of chloroplast genomes [14,15], small RNAs [16-19], and transcriptomes of organisms with [20-22] or without [23] extensive genomic sequence information.

These Next Generation (NG) sequencing methods promise a cost-effective means of either deeply sampling or fully sequencing an organism's transcriptome, with even small experiments tagging a very large number of expressed genes. However, prior transcriptome sequencing studies have been largely exploratory, only hinting at the potential for NG transcriptome sequencing at different scales. There is a great need for quantitative studies and analysis tools that help investigators optimally design NG sequencing experiments to address specific goals.

A complete solution to this problem would involve realistic models for each technology, accounting for the cost of library generation and data collection, the characteristics of cDNA libraries, transcript abundance distributions, read length distributions, and the error rates in sequence generation and assembly. The present study focuses on the first four of these issues to provide estimates of theoretical coverage of complex transcriptomes with varying scales and types of DNA sequencing experiments. In earlier publications [24,25], we developed a robust simulation approach to model traditional capillary transcriptome sequencing, which incorporates distributions of the relative start site of cDNA sequences as a function of cDNA length, the read length distribution, and the transcript abundance distribution. We have now adapted this simulation approach to model the specific characteristics of NG sequencing. The results from this study should help researchers working with these new and exciting technologies.

The present study has several goals. First, we report empirical comparisons of 454 pyrosequencing and capillary-based transcriptome sequencing from the model plant, *Arabidopsis thaliana*, and two non-model plant species, the basal eudicot *Eschscholzia californica *(California poppy) and the magnoliid *Persea americana *(avocado). We use these results to examine the effects of library preparation procedures, specifically, normalized versus non-normalized and random versus oligo-dT primed libraries. We then introduce a simulation approach, based on the GS20 sequencing results, to predict the outcome of additional GS20 transcriptome sequencing experiments while accounting for critical features in cDNA library construction. We then use the GS20 simulation results to extrapolate results for 454FLX and Solexa platforms, in order to estimate technology-specific sequencing characteristics. Finally, we report on simulated experiments aimed at characterizing the optimal mixture of methods for most complete and cost-effective transcriptome sequencing with one or more sequencing technologies.

## Results

### Next Generation Transcriptome sequencing of *Arabidopsis *floral tissue

A half plate of GS20 sequencing from an *Arabidopsis *random-primed cDNA library generated 134,791 reads totalling 13.8 MB with an average length of 102.2 bp. The reads were assembled into 82,281 unigenes, which included 8,188 contigs with an average length of 147 bp and 74,093 singleton reads (Table 1). We mapped 122,344 (90.8%) reads to the TAIR 7 *Arabidopsis *genome annotation (Table 2 and see Methods). Of the total mapped reads, 88.7% were located within 15,539 genic regions and 2.1% were located in intergenic regions. Within the genic regions, 119,518 (88.7%) reads mapped exactly to known exons, while 1,117 (0.8%) and 11,524 (8.6%) reads mapped to introns and intron/exon boundaries, respectively. Also, 3,066 (2.3%) of the reads included in the genic regions extended current boundaries of known genes while 302 reads combined two annotated genes or marked areas of the genome with overlapping genes. There were 12,447 (6.7%) reads that did not have a significant BLASTn match to any location within the genome. There were 1,085 genes that had more than 20 reads per locus, and the 10 most highly expressed genes (Table 3), included two subunits of the photosynthetic protein RuBisCo, as well as *TASTY*, *TGG1*, and *PDF1*. These "top ten" transcripts had read counts ranging from 190 to 586 reads with the RuBisCO small subunit 1A being most highly represented. At this shallow sequencing depth, 2 non-overlapping contigs, with lengths of 357 and 240 bp, mapped to the RuBisCO small subunit 1A gene.

**Table 1:** Sequencing statistics of analyzed libraries.

	Ath (random)		Pam (normal)		Eca (oligo-dT)		Eca (random)		Eca (combined)	
**Type**	**n**		**n**		**n**		**n**		**n**	

**Reads**	134,791	102.2	269,057	85.9	251,716	98.9	307,836	98.2	559,552	98.6

**Contigs**	8,188	147.0	22,303	107.3	18,339	148.5	14,242	146.9	30,603	159.1

**Singletons**	74,093	101.6	211,882	85.0	64,931	99.9	61,031	99.1	89,982	99.5

**Unigenes**	82,271	106.1	234,185	90.6	83,270	107.7	75,273	105.1	120,585	106.9

**MB**	13.8		23.1		24.9		30.2		55.1	

Read, Contig, Singleton, and Unigene Counts (n), mean sequence lengths (), and total amount of sequence data (MB) for 454 GS20 libraries analyzed. Species codes are Ath (*Arabidopsis thaliana*), Pam (*Persea americana*, avocado), Eca (*Eschscholzia californica*, California poppy). cDNA library production method indicated in parentheses. Read lengths based on number of Q20 equivalent bases produced, after trimming and cleaning with the program seqclean http://compbio.dfci.harvard.edu/tgi/software/; normalized library original read mean length was 100.1 prior to trimming normalization adapter.

**Table 2:** *Arabidopsis *454 reads mapped to the annotated genome.

Sequence Type	Reads	Genes	Total (%)
**Genes**	119,518	15,539	88.7

**Exon**	103,509	14,754	76.8

**Intron**	1,117	877	0.8

**Intron/Exon**	11,524	5,973	8.6

**Extended UTR**	3,066	1,635	2.3

**Overlapped Genes**	302	177	0.2

**Intergenic**	2,826	2,096	2.1

**No Hit**	12,447		9.2

**Total**	134,791		100.0

All 454 reads were mapped (BLAST-n, default parameters) to the genome. TAIR XML files were parsed to obtain exon structure and location within the genome. Percentages were calculated for each class of sequence type. The number of genes does not equal the summation of gene components because there are some genes that are hit by multiple reads in different sections of the gene. The percent for each gene component is the percent of total reads.

**Table 3:** Top 10 most frequently detected unigenes in 454 cDNA libraries of *Arabidopsis*, *Eschscholzia*, and *Persea*.

Library	Contig	Len	Reads	Cov	AGI	Len	Evalue	Annotation
Ath-rand	08061	357	586	34.8	AT1G67090	1025	0.0	RuBisCO small subunit 1A (RBCS-1A) (ATS1A)

Ath-rand	00035	1326	541	96.8	AT1G54040	1370	0.0	TASTY, ESP (EPITHIOSPECIFIER PROTEIN)

Ath-rand	08724	1653	391	90.0	AT5G26000	1836	0.0	TGG1 (THIOGLUCOSIDE GLUCOHYDROLASE1)

Ath-rand	08295	1175	278	94.6	AT2G42840	1242	0.0	PDF1 (PROTODERMAL FACTOR 1)

Ath-rand	08670	310	258	31.5	AT5G38410	984	4e-175	RuBisCO small subunit 3B (RBCS-3B) (ATS3B)

Ath-rand	00011	240	229	23.4	AT1G67090	1025	9e-43	RuBisCO small subunit 1A (RBCS-1A) (ATS1A)

Ath-rand	00660	640	219	76.9	AT2G21660	832	2e-157	ATGRP7 (Cold, Circadian Rhythm, RNA Binding 2)

Ath-rand	07960	927	215	52.6	AT5G60390	1764	0.0	elongation factor 1-alpha/EF-1-alpha

Ath-rand	04760	1157	206	82.3	AT3G12145	1406	0.0	FLR1 (FLOR1); enzyme inhibitor

Ath-rand	08550	373	190	100	ATCG00220	105	3e-53	PSBM, PSII low MW protein

Eca-oligo	19682	387	850	83.2	AT5G39170	465	2e-7	Unknown protein

Eca-oligo	19707	2089	784	100	AT1G70370	1878	0	BURP domain-containing protein/polygalacturonase

Eca-oligo	18128	151	707	10.0	AT3G47550	1505	0.02	C3HC4-type RING finger family protein

Eca-oligo	19695	308	678	100	AT5G52160	288	1e-15	protease inhibitor/seed storage/lipid transfer protein

Eca-oligo	19793	940	608	100	AT2G36830	753	6e-102	GAMMA-TIP (Tonoplast intrinsic protein gamma)

Eca-oligo	18734	849	485	100	AT3G16640	504	7e-52	TCTP (Translationally Controlled Tumor Protein)

Eca-oligo	00048	2823	450	80.0	AT5G35750	3528	0	AHK2 (Arabidopsis Histidine Kinsase 2)

Eca-oligo	18697	144	450	24.7	AT4G06746	584	0.31	RAP2.9 (related to AP2 9); transcription factor

Eca-oligo	19623	2638	421	81.4	AT2G01830	3240	0	WOL (CYTOKININ RESPONSE 1)

Eca-oligo	19622	120	415	6.4	AT1G23800	1866	1	ALDH2B7 (Aldehyde dehydrogenase 2B7)

Eca-rand	15341	109	4296	6.9	AT4G03930	1575	0.23	Pectinesterase

Eca-rand	15345	162	4274	12.0	AT3G59430	1353	0.33	Unknown protein

Eca-rand	15162	315	852	19.7	AT5G26670	1596	0.18	Pectinacetylesterase, putative

Eca-rand	15258	606	726	53.3	AT3G12340	1137	0.1	FK506 binding/peptidyl-prolyl cis-trans isomerase

Eca-rand	14312	182	682	56.2	ATMG00030	324	2e-77	ORF107A

Eca-rand	15290	2020	674	100	AT1G70370	1878	0	BURP domain-containing protein/polygalacturonase

Eca-rand	15208	1052	514	100	AT2G36830	753	7e-102	GAMMA-TIP (Tonoplast intrinsic protein gamma)

Eca-rand	14424	2660	480	75.4	AT5G34750	3528	2e-162	AHK2 (ARABIDOPSIS HISTIDINE KINASE 2)

Eca-rand	15320	1146	437	48.3	AT5G02500	2373	0	HSC70-1 (heat shock cognate 70 kDa protein 1)

Eca-rand	15269	304	417	5.8	AT2G47410	5221	0.2	Nucleotide binding

Pam-norm	15603	133	37	9.8	AT1G59830	1357	0.005	PP2A-1 (protein phosphatase 2A-2)

Pam-norm	18074	139	32	10.4	AT1G14270	1343	0.3	CAAX amino terminal protease family protein

Pam-norm	8473	176	27	10.4	AT4G17890	1688	0.1	AGD8, UBP20 (Ubiquitin-specific Protease 20)

Pam-norm	14132	213	26	7.3	AT2G40820	2907	1.9	Proline-rich family protein

Pam-norm	15140	237	26	48.5	AT2G41430	489	2e-13	ERD15 (Early Responsive To Dehydration 15)

Pam-norm	4395	144	25	6.4	AT1G45545	2259	0.08	Similar to unknown protein

Pam-norm	15762	102	24	3.5	AT1G01950	2901	0.2	Armadillo/beta-catenin repeat family protein

Pam-norm	10833	112	20	6.4	AT3G03640	1747	0.001	GLUC (Beta-glucosidase homolog)

Pam-norm	18760	253	19	59.0	AT4G14270	429	2e-04	Protein containing PAM2 motif

Pam-norm	18306	208	18	48.5	AT4G14270	429	8e-05	Protein containing PAM2 motif

Unigenes from each library, *Arabidopsis *flower bud random-primed (Ath-rand), *Eschscholzia *flower bud oligo-dT (Eca-oligo) and random-primed (Eca-rand), and *Persea americana *normalized flower bud (Pam-norm), were mapped to the annotated TAIR cDNA and protein datasets using BLASTx (e-5 cutoff). Column headers are contig name (Contig), contig length (Len), number of reads per contig (Reads), percent coverage (Cov), *Arabidopsis *best hit gene identifier (AGI), annotation (Annotation), and E-value. Ribosomal RNA and contaminants such as putative endophytes removed from this list. Refer to Additional file 1 for detailed BLAST results.

Despite low overall transcriptome coverage, one-half plate of *Arabidopsis *GS20 sequence data returned 27 fully sequenced cDNAs, as well as 292, 628, and 1008 genes at 90%, 80%, and 70% coverage, respectively. These results demonstrate that nominal amounts of 454 sequencing can generate complete or nearly complete sequences for an appreciable number of genes, especially those that are small and highly expressed. Another very promising result is the improved annotation of genes for both model and non-model species. For example, although the *Arabidopsis *genome has been largely sequenced since 2000 [26], the half plate of GS20 extended the untranslated regions (UTRs) of roughly 3,066 genes and mapped new transcript boundaries of 8,662 genic regions. These regions are possibly new splice variants of previously annotated genes. Finally, 2,826 transcripts were mapped to 2,096 unique intergenic regions. These transcripts might represent un-annotated protein-coding genes or non-coding RNA sequences that have not previously been sampled in traditional cDNA libaries.

### Transcriptome sequencing of *Eschscholzia californica *using oligo-dT and random-primed libraries

Two full plates (over 559,000 total reads) of GS20 sequencing was performed on the emerging model basal eudicot, *Eschscholzia californica *[27,28], including one plate from a 454 library of oligo-dT primed cDNA and one plate from a 454 library of random hexamer-primed cDNA. The library of oligo-dT primed cDNA generated 251,716 reads totalling 24.9 MB with an average length of 98.9 bp. The reads assembled into 83,270 unigenes, including 18,339 contigs with an average length of 148.5 bp and 64,931 singletons (Table 1). The library of random-primed cDNA generated 307,836 reads totalling 30.2 MB with an average length of 98.2 bp. The reads assembled into 75,273 unigenes, including 14,242 contigs with an average length of 146.9 bp and 61,031 singleton reads (Table 1). Finally, we assembled both plates, which resulted in 120,585 unigenes, including 30,603 contigs with an average length of 157.0 bp and 89,892 singleton reads (Table 1).

**Figure 1:**
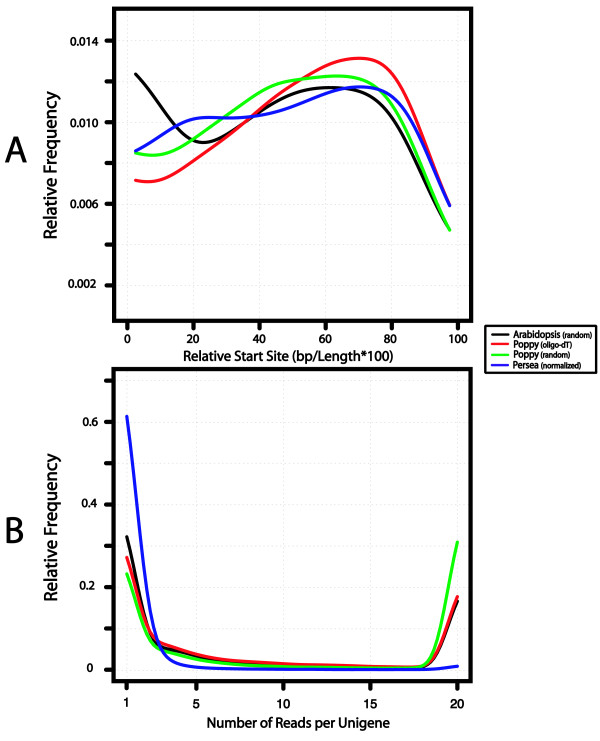
**Distributions of relative start sites and number of reads per gene**. A. Start site distributions of 454 sequences for each species in this study including random, oligo-dT, and normalized oligo-dT libraries. Sequencing start sites are calculated as the start position, defined by BLASTn (*Arabidopsis*) or BLASTx (*Eschscholzia*, *Persea*) hit divided by the cDNA or protein length and expressed as percentage of the gene length. B. Distribution of the number of reads from each library mapped to an *Arabidopsis *gene, defined by best BLASTn or BLASTx hit of each read to the TAIR genes. Species abbreviations are ATH (Arabidopsis thaliana), ECA (*Eschscholzia californica*), and PAM (*Persea americana*).

As expected, the most obvious difference between the oligo-dT and random-primed cDNA sequences was the representation of rRNA genes. Additional rounds of mRNA purification, however, could have reduced the level of rRNA "contamination". We also examined the relative start positions of the reads from each library by mapping the reads to the proteome of *Arabidopsis *(Figure 1A). The relative start positions are defined as the start position of the best *Arabidopsis *HSP divided by the length of the best protein match. As expected, the oligo-dT library had a greater 3' bias than the random primed library. The unigenes from both libraries mapped to 6,498 unique *Arabidopsis *genes, with 4,066 of the transcripts found in both. The level of redundancy observed between these two plates (just 62.6%) suggests that many more genes would be discovered with additional sequencing.

### Transcriptome sequencing in a normalized library of *Persea americana*

One plate of GS20 sequencing was performed on a normalized library for *Persea americana*, an emerging model for the magnoliids [29]. The plate generated 298,055 reads totalling 29.8 MB with an average length of 100.1 bp. We then trimmed the adaptors used in the normalization step, which reduced the total number of reads to 269,057 with an average sequence length of 85.9 bp. Trimming the adaptors reduced the total amount of sequence by more than 6 MB, bringing the total to 23.1 MB. The reads assembled into 234,185 unigenes, including 22,303 contigs with an average length of 107.3 bp and 211,882 singleton reads (Table 1).

To determine the success of the normalization step, we plotted the relative frequency of the number of reads per gene, using *Arabidopsis *as a reference (Figure 1B). Compared to the other library methods used in this study, the normalized *Persea *library (solid blue line) contained the largest number of genes with fewer than five reads per gene and the fewest number of genes with more than 5 reads per gene. The gene with the highest number of mapped reads was a protein phosphatase with 37 reads. In contrast, the most highly represented genes in the poppy non-normalized libraries had over 1000 reads mapping to specific *Arabidopsis *genes. Hence, the normalization step was successful. Note that the *Persea *library, constructed using the Trimmer-Direct Kit (Evrogen) with amplification of full-length cDNAs (Clontech's SMART technology), also has the least amount of 3' bias in read start positions (Figure 1A).

### Correlation of observed *Arabidopsis *transcript frequencies with microarray data

**Figure 2:**
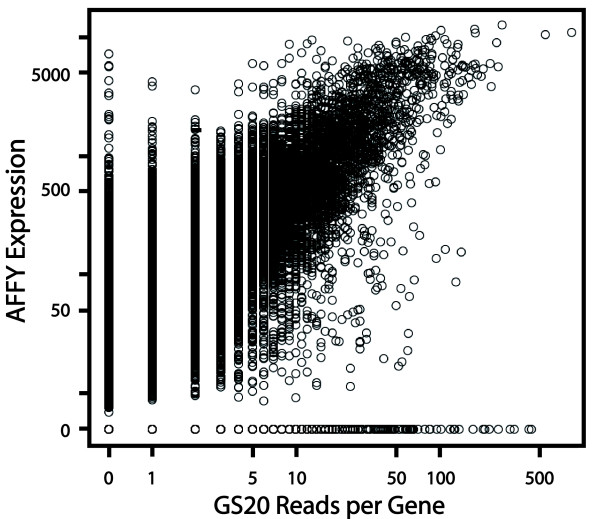
**Correlation of gene expression with number of transcripts**. Linear Regression comparing number of 454 reads with Affymetrix (AFFY) gene chip expression values for *Arabidopsis *young inflorescence. Each symbol represents a single gene, with many genes having overlapping counts. Correlation between the two measures of gene expression is highly significant (r = 0.67, r2 = 0.444, p < 0.0001).

Of the 21,707 genes included on the *Arabidopsis *Affymetrix (AFFY) microarray, 13,790 had at least one read mapped to its cDNA sequence. For these genes, we used AFFY microarray expression values generated from inflorescence tissue in the same *A. thaliana *ecotype [30] to compare with the number of 454 reads for each gene. The comparison revealed that 1,907 genes that were detected above normalized expression level 50 with the AFFY chip were not detected in the 454 sequences, while 1,375 genes were detected in 454 reads, but were below expression level 50 with AFFY data (a common cutoff for reliable detection with the AFFY system). An additional 1,717 genes detected by 454 reads were not included as probes on the AFFY gene chip. A moderate correlation was observed between microarray expression values and number of 454 reads (Figure 2 with r = 0.67, r^2 ^= 0.444, p < 0.0001).

### Next Generation transcriptome simulation study

A primary goal of large-scale transcriptome sequencing is to identify and obtain full-length sequences of all of the expressed genes in an organism or tissue. A researcher will typically begin with RNAs isolated from a tissue of interest or a collection of tissues from the entire organism. The researcher may use tissue from a particular developmental stage or assay gene expression under a range of experimental conditions (e.g., light/temperature/water/nutrient stress, gene knock out). Each of the new NG technologies (e.g., 454-GS20/FLX, Solexa) produces data with characteristics that can be evaluated and compared to each other and traditional capillary sequencing.

In order to predict the expected outcomes of varied amounts of sequencing effort using a blend of technologies, we developed a predictive model based on the simulation engine of ESTstat [24,25]. Inputs to the model include four distribution profiles that reflect information about the cDNA library or sequencing technology: 1) the transcript abundance profile, a transcriptome-specific frequency distribution of the number of tags of different genes in the entire transcriptome, 2) the distribution of cDNA lengths 3) the distribution of sequencing start sites, and 4) the distribution of read lengths after removal of vector and low quality data. The first three of these reflect library specific features, while the fourth is mostly dependent upon the sequencing technology. The ESTstat simulation model has been tested under a variety of situations and found to robustly predict the outcomes of future sequencing experiments. Although ESTstat can estimate and correct assembly errors *in silico *without reference to a known genome sequence, we were able to map each read to its known location on the *Arabidopsis *genome to assess and correct assembly error.

**Figure 3:**
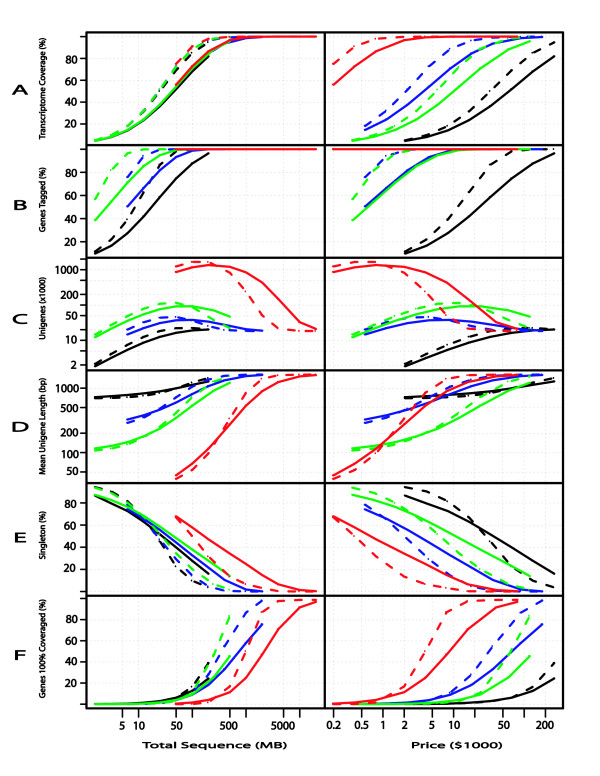
**Simulation results for different Next Generation sequencing technologies**. Simulation results illustrating predicted outcomes for different transcriptome sequencing technologies with a complex library expressing ca. 18,000 genes. Left column illustrates predicted outcomes as a function of MB of sequence; right column gives predicted outcomes as a function of estimated sequencing cost (see text for cost assumptions, which do not include varied costs for RNA isolation and library preparation). Each simulated data set was used to calculate: A) percent of transcriptome sequenced with at least one read and not necessarily in one contiguous sequence, B) number of genes tagged, C) number of unigenes obtained, D) mean unigene length (bp), E) percent of reads that are singleton sequences, and F) the number of genes with 100% coverage. Each technology is represented by a different line color, with solid lines indicating non-normalized libraries and dashed lines indicating theoretically perfectly normalized libraries. EST5 = 5' capillary sequence (black); GS20 = 454 GS20 (green); GSFLX = 454 GSFLX (blue); SOL = Solexa (red). The following prices (per MB) were used in the calculations: EST5 ($1330), GS20 ($240), GSFLX ($90), and SOL ($4). For several of the measures, the Solexa result is hidden under the topmost line. Additional details provided in text.

We used the results from our GS20 sequencing to simulate different levels of sequencing coverage for each of the NG and capillary technologies. For each technology, we considered both non-normalized and perfectly normalized libraries, in which the expression level of every gene is made identical. Actual normalization experiments should therefore fall somewhere between non-normalized and perfectly normalized, depending on the normalization method, RNA quality, and success of the normalization procedure (see Materials and Methods for more detail). We used the following parameters to help evaluate the different sequencing platforms: transcriptome coverage, percentage of all expressed genes that were tagged, percentage of singletons, number of unigenes, mean unigene length, and the percentage of all expressed genes that were sequenced completely (i.e. 100% covered; Figures 3A, 3B, 3C, 3D, 3E, and 3F).

*Transcriptome coverage *(Figure 3A) is a direct indicator of the sequencing depth and breadth of sequence data relative to the sample transcriptome. We define the transcriptome coverage as the total non-redundant number of bases from sampled genes that are included in at least one EST, divided by the sum of cDNA lengths for all expressed genes (including both detected and undetected genes in the transcriptome). In this study, the 15,276 detected genes and randomly sampled 3,007 undetected genes (estimated using ESTstat, see Materials and Methods) sum to 18,283 genes, with an expected total cDNA length of 29.8 MB. The transcriptome coverage, as a function of the total number of sequenced bases (MB), differs only slightly for all technologies. However, when the amount of sequence is low (1–500 MB), the transcriptome coverage is greater in the normalized libraries (dashed lines) compared to the non-normalized libraries (solid lines) for each technology. Theoretically, perfect normalization will equalize the level of expression for all genes, without any other impact on library quality, and thus will increase the coverage of genes that are randomly sampled. Using the distributions of cDNA length, read length, and sequencing start sites obtained in these experiments, we estimate that traditional 5' capillary sequencing of a non-normalized library will cover approximately 14%, 52%, and 82% of the transcriptome with 6.25, 50, and 200 MB of sequencing, respectively. For a normalized library, the percentage increases to 18%, 69%, and 95% with the same amounts of sequence. The same pattern was observed for the NG technologies but with higher levels of transcriptome coverage. For example, the GS20 technology is estimated to cover 15%, 54%, and 88% of the transcriptome for a non-normalized library and 18.2%, 72%, and 98% of the transcriptome for a normalized library at 6.25, 50, and 200 MB of sequencing. The lower coverage of capillary-based EST sequencing given the same number of sequenced bases is attributed to biases implicit in the cDNA cloning process. The FLX is estimated to cover 15%, 54%, and 88% for the non-normalized library and 18%, 72%, 98% for a normalized library at the same intervals. Finally, the Solexa platform is estimated to cover 55% and 87% for the non-normalized library and 75% and 98% for the normalized library for 50 and 200 MB, respectively. Given that one plate of sequence data from the Solexa platform is estimated at 1,000 MB, we chose 50 MB (1/20 of a plate) as the first interval to be simulated, and we excluded all intervals less than 50 MB.

Transcriptome coverage differs substantially among the various technologies at the same cost. However, the cost used in this analysis refers only to the actual sequencing costs and not the pre-processing costs such as library preparation and normalization. The Solexa platform rapidly approaches 100% coverage primarily because the cost of sequencing is substantially smaller per MB (simulations for Solexa were based on $4000/plate at 1,000 MB/plate). Solexa is followed by GS20, FLX, and conventional EST sequences. It is estimated that traditional capillary sequencing would reach 100% transcriptome coverage at more than 200 MB and at a cost of over $200,000. While Solexa sequencing is the most economical technology for deep coverage of transcriptomes, *de novo *assembly of short Solexa sequences for non-model species remains an unresolved challenge.

A second indicator of the depth of transcriptome sequencing is the *percentage of genes tagged *(Figure 3B). A gene is considered tagged if it has been sampled with at least one read. The percentage of genes tagged increases with both amount of sequencing and price. For a non-normalized traditional library, we estimate that 27%, 75%, and 96% of the genes will be tagged in our sample transcriptome with 6.25, 50, and 200 MB of sequencing. For a normalized library, the percentage increases to 39%, 98%, and 100% with the same amounts of sequence. As expected, this percentage increases when the sequencing is done with any of the NG technologies. The cost of gene tagging also differs substantially among the various sequencing technologies. The Solexa platform tags essentially 100% of the expressed genes with less than one plate of sequence ($4000). Solexa is followed by GS20, FLX, and conventional EST sequences. Capillary sequencing would approach 100% genes tagged at more than 200 MB and over $200,000.

The *number of unigenes *(Figure 3C) – including singletons and contigs – has typically been used to estimate the number of transcribed genes in a tissue. With small amounts of sequencing, the number of unigenes is similar to the number of sequences, but with more sequencing multiple reads are observed for each gene (increasing redundancy), and the rate of discovery for new genes falls off. At a particular point in the sequencing process (peaks in Figure 3C), the number of unigenes will begin to decrease as disconnected reads coalesce into contigs covering entire genes, and eventually the unigene number approaches the number of genes expressed in the library. The rate at which multiple reads for a gene coalesce into a single contig is a function of read length. With the capillary technology, each read is large compared to the NG reads. With a non-normalized library similar to the model library, we will reach the peak unigene number at more than 200 MB of sequencing. With a normalized library, we reach the peak at approximately 100 MB and decrease gradually with an additional 100 MB of sequence. However, we still do not reach the estimated 18,000 genes expressed in the *Arabidopsis *floral library. For the FLX technology, the maximum number of unigenes occurs at roughly 100 MB and 50 MB for the non-normalized and normalized libraries, respectively. However, because the FLX sequences are two to three times shorter than the traditional sequences, the peak is reached with roughly double the number of unigenes (38,000 and 46,000, respectively). For the GS20 platform, the peaks occur at nearly the same levels (approximately 100 MB) as the FLX platform, but since these reads are half as long as FLX reads, the GS20 produces more than twice the number of unigenes (92,000 and 115,000) for both library types. The Solexa platform produces many more unigenes at all levels of sequencing and the peak occurs at approximately 200 MB for both library types (1.3 and 1.7 million reads).

The *mean unigene length *(Figure 3D) is an important statistic if the goal of the transcriptome sequences is to perform multi-gene phylogenetic or molecular evolutionary analyses. In this case, researchers would like full-length sequences for many expressed genes, not just small fragments of expressed genes. In the *Arabidopsis *genome, the average transcript length is approximately 1,500 bp (1,436 for all transcripts and 1,628 bp for only the transcripts predicted to be expressed in this library). Therefore, a researcher would like to sequence enough of a library to produce contiguous sequences with average lengths of all genes in the library. We calculated the unigene length in two different ways. First, we used the mean length of all unigenes, although this estimate lowers the mean length for the shorter sequences in the NG technologies. Second, we calculated the mean length of only the longest unigenes for each gene (Figure 3D). All NG technology and library type combinations require greater depth of sequencing to reach the same level as its traditional counterpart. When we examine the mean unigene length in relation to price, the traditional sequencing produces the longest unigenes until approximately $5,000 worth of sequencing. This is approximately 4–5 MB of capillary sequencing and 6,000–8,000 reads. At this point, the NG technologies begin to generate enough sequences to assemble longer unigenes at a lower cost.

The *percentage of singleton reads *(Figure 3E) reflects sequencing depth and the likelihood that a given read will assemble to form a contig with other reads. A singleton is defined as a single read that does not contain enough overlap in length to be combined with other reads from the same transcribed gene. The percentage of singletons is also inversely proportional to the levels of redundancy in the library. Therefore, additional sequencing usually reduces the percentage of singletons. This is the case for capillary sequencing, where the percentages of singletons are 73%, 40%, and 16% for non-normalized and 81%, 23%, and 4% for normalized libraries at the 6.25, 50, and 200 MB levels, respectively. For the GS20, these values change to 76%, 48%, and 25% for non-normalized libraries and 80%, 34%, and 7% for normalized libraries at the same levels. For the FLX, the percentage of singletons changes to 74%, 44%, and 22% for non-normalized and to 78%, 29%, and 5% for normalized libraries at the same levels. Finally, for Solexa, the percentage of singletons is predicted to be around 68%, 47%, and 25% for non-normalized and 67%, 32%, and 7% for normalized libraries at the 50, 200, and 1000 MB sequence intervals, respectively.

The final parameter used to evaluate and compare the technologies is the *percentage of genes with 100% coverage *(Figure 3F). As with mean unigene length, gene coverage can be calculated using all of the unigenes per gene, or by using only the longest unigene. The smaller reads from the NG technologies might cover all the regions within a gene. However, many of the reads for a gene will not have sufficient overlap to assemble into a contiguous sequence. Although we calculated both estimates, we use the percentage of gene coverage based on the longest unigene for comparisons to other platforms. In relation to amount of sequencing (MB), the capillary, GS20, and FLX technologies have similar percentages. The Solexa platform requires more data (MB of sequencing) to fully sequence a similar number of genes. For example, the FLX generates unigenes that completely cover roughly 18% and 58% of the total genes with 200 MB and 1000 MB of sequence data. The same amounts of Solexa sequencing would fully sequence 4% and 25% of the genes. However, the FLX experiment would cost approximately $18,000 and $90,000, whereas the Solexa data could be generated for roughly $800 and $4,000. Finally, with capillary sequencing, 200 MB would need to be sequenced at $250K to fully cover 25% of the genes.

### Combinations of traditional and NG sequencing

Analyses of genome sequencing projects suggest that optimal genome assemblies can be obtained through a combination of traditional and NG technologies [11]. In order to investigate the combination of these new technologies for transcriptome sequencing, we examined the addition of NG sequences to traditional capillary sequences (Figures 3A, 3B, and 3C) and the combinations of NG sequences alone (Figures 3D, 3E, and 3F). All of the indicators from the previous section dramatically improved with the addition of small amounts of NG sequences. Among the various combinations of technologies, there is little difference in most of the indicators used in the previous section. For example, the percentage of genes tagged approaches 100% with very small amounts of NG sequences. Therefore, to evaluate the various combinations of technologies, we compared three of the statistics described above: mean unigene length, transcriptome coverage, and percent of genes 100% covered.

**Figure 4:**
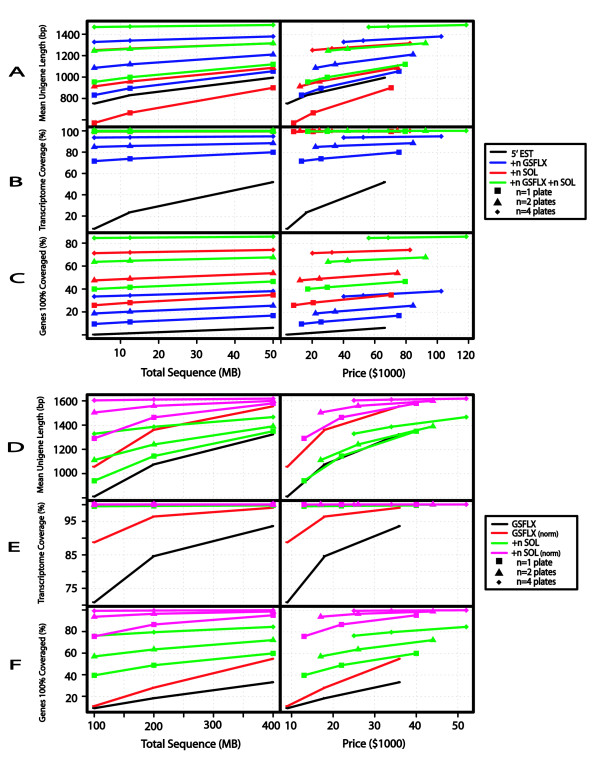
**Simulation results for combinations of Next Generation sequencing technologies**. Illustrating predicted outcomes from combined sequencing technologies with a complex library expressing ca. 18,000 genes. A-C) Combinations include 3.125, 12.5, or 50 MB 5' Sanger sequencing plus 0 to 4 plates of GSFLX and/or Solexa sequence. Each technology or combination of technologies is represented by a different line color, with black indicating Sanger alone, and blue, red, and green lines indicating the addition of GSFLX, Solexa, and GSFLX+Solexa, respectively. The square (n = 1), triangle (n = 2), and diamond (n = 4) shaped points on each line indicate the number of plates added for each technology. Results shown for non-normalized libraries. A) Mean length of longest Unigene per gene (bp), B) Transcriptome Coverage (%), and C) Number of Genes with 100% coverage as a function of total sequence (MB, left panel) and estimated sequencing cost ($1000, right panel) for the different technologies and combinations of technologies. Abbreviations and cost functions are as described in Figures 3D, 3E, and 3F) Combinations include 100, 200, or 400 MB of GSFLX non-normalized or normalized sequencing plus 0 to 4 plates of Solexa sequence. Each technology or combination of technologies is represented by a different line color, with black and red lines indicating GSFLX alone with non-normalized and normalized libraries, respectively. Green and pink lines indicate the addition of Solexa non-normalized and normalized sequences, respectively. The square (n = 1), triangle (n = 2), and diamond (n = 4) shaped points on each line indicate the number of plates added for each technology. D) Mean length of longest Unigene per gene (bp), E) Transcriptome Coverage (%), and F) Number of Genes with 100% coverage as a function of total sequence (MB, left panel) and estimated sequencing cost ($1000, right panel) for the different technologies.

The addition of NG sequences to traditional capillary sequences increased each of these three indicators at most sequence increments (Figures 4A, 4B, and 4C). Only the addition of one plate of Solexa and all GS20 plate increments decreased the mean unigene length (Figure 4A). The addition of four plates of FLX increased the mean unigene length to 1327 and 1380 bp with 3.25 and 50 MB and of traditional sequences, respectively. At these same increments, transcriptome coverage would increase from 94% to 95% (Figure 4B), while the percent of genes 100% covered would increase from 33% to 38% (Figure 4C). The addition of this amount of FLX would increase the total cost of sequencing from $40K to $102,000. However, sequencing only four plates of FLX, assuming perfect assembly, could in theory generate 1323-bp unigenes at under $40,000, with approximately 94% transcriptome coverage and covering 37% of the genes 100% covered. Adding four plates of Solexa to four plates of FLX would generate 1466 bp unigenes at just over $50,000 (Figure 4D). This amount of sequencing would cover 100% of the transcriptome (Figure 4E) and fully sequence 84% of the genes (Figure 4F). Under these conditions, the primary advantage of including Sanger sequences would be the improvement of assembly through the inclusion of long individual reads, and simplification of downstream experiments with physical clones.

### ESTcalc: A simulation calculator for NG transcriptome sequencing experiments http://fgp.huck.psu.edu/NG_Sims/ngsim.pl

With sequencing technologies rapidly advancing, researchers will wish to predict the cost and potential outcomes of diverse transcriptome sequencing projects under a wide range of initial assumptions. We have constructed ESTcalc, an online webtool, which allows users to explore the results of this study by specifying individualized costs and sequencing characteristics. Users can choose a single sequencing method (5' Sanger sequencing, GS20, GSFLX, or Solexa), perfectly normalized or non-normalized libraries, and varied amounts of sequencing and read lengths to predict many of the same parameters used in this study. User-defined costs can include both fixed (e.g., cost of obtaining libraries) and per unit sequencing costs, with default costs the same as used in our study. ESTcalc will extrapolate from the closest treatments examined in our simulation study, and give outcomes such as the project cost, predicted number of unigenes, unigene length, transcriptome coverage, and related statistics presented earlier in this study. Combinations of sequencing strategies, such as normalized plus non-normalized libraries, or combinations of different technologies, can also be examined under the same range of combinations used in our study. Additional combinations, including parameter sets for SOLiD sequencing, will be added to ESTcalc as they are obtained in ongoing data analyses.

## Discussion

### Next Generation transcriptome sequencing

Next Generation sequencing has great potential for accurate transcriptome characterization because of the large amount of data obtained at considerably lower costs compared to traditional methods. Although the cost of traditional sequencing (over $1000/MB) has continued to decrease over the last decade, the lower cost of NG sequencing ($250/MB for GS20, $90/MB for FLX, and $5/MB for Solexa) will dramatically improve transcriptome sequencing in future research. The overall yield and value of NG sequencing is evident in the amount of sequence data obtained in each run. We identified a large number of uniquely tagged gene sequences in each of our three cDNA libraries (*Arabidopsis*, poppy, and *Persea*). With only a small amount of sequencing (one-half plate on the GS20) in *Arabidopsis*, we tagged more than 15,000 genes and completely or nearly completely sequenced several hundred of the highly expressed genes. Even with a very modest amount of data by NG sequencing standards, many of these sequences extended the annotated untranslated regions (UTRs) and redefined intron/exon boundaries, including evidence of alternative splicing. We also identified more than 2,000 transcripts that were not previously annotated in the *Arabidopsis *genome. These may define new genes or transcribed non-coding regions such as miRNA or other small RNA. In any event, these results illustrate the utility of NG transcriptome sequencing for genome annotation.

**Figure 5:**
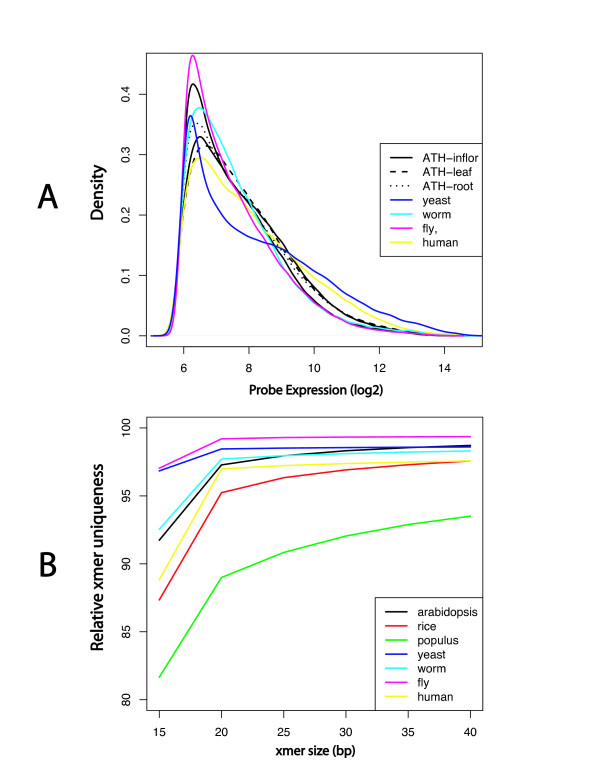
**Probe expression distributions and relative uniqueness of varying x-mer sizes**. A. Probe expression distribution for different tissues of Arabidopsis and various other species, as indicated. Data shown are the smoothed histograms of the log2 (perfect match expression) values for each sample taken from microarray datasets (see text). Datasets are adjusted to the same fifth percentile. B. Relative uniqueness of varying x-mer sizes in full cDNA collections from several sequenced genomes. For each species, specified by a different colored line, we determined all DNA x-mers of various sizes (x = 15, 20, 25, 30, 35, or 40 bp). Relative x-mer uniqueness is number of x-mers unique to only one gene divided by the total number of x-mers in the transcriptome. The level of uniqueness increases with size of x-mer, and varies with the organism.

Our data were limited to *Arabidopsis *inflorescence, and there are likely to be differences in experimental outcomes using different organisms and tissue. To assess the similarity of the *Arabidopsis *inflorescence transcriptome with other transcriptomes, we considered the distribution of intensities of the perfect match probes from several Affymetrix experiments involving various tissues and organisms. We examined data from *Arabidopsis *inflorescence, leaf, and root on ATH1 arrays [30], human skeletal muscle [31] on the hgu95av2 array, *Caenorhabditis elegans *(whole worm) [32] on the *C. elegans *array, *Drosophila melanogaster *(whole fly) [33], and *Saccharomyces cerevisiae *(yeast) on ammonium sulfite nitrogen source [34]. Small differences are observed in the expression profiles, consistent with some samples having different proportions of genes expressed more or less highly, but overall, the distribution of expression intensities is very similar for all of the samples (Figure 5A). These differences among samples are on the same scale, and sometimes smaller than, the variations seen among replicate samples from *Arabidopsis *inflorescence. Because the tissue-specific expression profile is the one method-independent input to the simulation model, we can expect similar predictions for transcriptomes from different sources.

### NG sequencing simulation studies and comparisons of platforms

Simulation studies help researchers predict outcomes of expensive or time consuming experiments that cannot be readily performed in the near term. For transcriptome sequencing, simulation studies have allowed researchers to conduct in silico experiments of systems that would be costly and time-consuming to do in the lab. We have developed a simulation approach to understand the advantages of each of the NG technologies in comparison with traditional capillary sequencing. Although all technologies eventually converge at similar points with regard to unigene length, transcriptome coverage, and percentage of genes fully sequenced (1,444 bp, 18,283 genes, and 100% for *Arabidopsis*), the NG technologies offer huge advances, most notably in the amount of sequence generated at considerably lower costs. Even though small NG experiments will tag a very large fraction of the transcriptome, it will commonly be in the form of thousands of disconnected fragments of genes, with relatively few full-length cDNAs. Thus, NG technologies are very effective for tagging sequences from fully sequenced species. However, researchers sequencing transcripts from novel species with few genomic resources or from species that are evolutionarily distant from a sequenced model organism might face several challenges when evaluating the data. The problems might become amplified with 25–30 bp reads generated by the Solexa system or other short-read platforms. The benefits of normalization are most evident in traditional sequencing, although some benefits, which include longer unigenes, are apparent in the NG technologies. However, the cost of normalization, and the potential for loss of closely related genes from the dataset, might outweigh the potential benefits.

Although NG sequencing does outperform traditional sequencing in many areas, the problems in assembly cannot be underestimated. Solexa and SOLiD sequences, currently less than 40 bp, will pose problems in assembly of unigenes, especially for short segments of genes that may be present in several genes. For example, only 81.7% of 15-mers are unique in the *Populus *transcriptome (PTR, Figure 5B). This leaves nearly 7 million 15-mers not unique to the *Populus *transcriptome, including 43,069 15-mers that are present in at least 10 different genes (results not shown). Until methods are developed to deal with this large fraction of sequence fragments that might lead to mis-assembled unigenes, researchers will not be able to use the Solexa or SOLiD technologies alone for transcriptome sequencing in non-model species. Research into genome assembly strategies with these short sequences with and without a reference genome is currently under investigation [35-37] and will hopefully become part of transcriptome assembly. The addition of Solexa sequences to longer NG sequences or traditional capillary sequences should help assemble larger unigenes. These longer sequences will have a much higher confidence in their uniqueness. The combination of technologies for transcriptome sequencing is analogous to genome shotgun sequencing which uses varying sizes of clones.

In order to evaluate the robustness of our simulator to both different organisms and tissues, we compared our simulated results against the poppy and avocado transcriptomes generated for this study and against two recently published 454 transcriptomes [22,23]. Vera et al. (2007) performed de novo assembly of 454-transcriptome sequences in the butterfly *Melitaea cinxia*. The RNAs were isolated from a genetically diverse pool of larvae, pupae, and adults. The authors sequenced two plates of GS20, and after trimming and cleaning, there were 518,079 reads (approximately 50 MB) with an average length of 110 bp. The reads assembled into 108,297 unigenes that included 59,945 singleton reads (55.4% singletons). The mean unigene length of 149 bp is the summation of 197 bp for all contigs plus the 110 bp average for the singleton reads. From our simulation results, we would predict roughly 91,000 unigenes, 48% singletons, and an average unigene length of 177 bp. Weber et. al sequenced 2 GS20 plates of cDNA derived from aboveground tissues of 8-day old light-grown Arabidopsis seedlings. The reads tagged an estimated 17,499 cDNAs, which is nearly identical to what our simulations would predict for that amount of GS20 data. For poppy, each of the observed assembly characteristics (Table 1) are very close to the predicted values for this amount of GS20 sequence. For example, the average unigene numbers for each of the poppy libraries are 83,370 and 75,273, which are very close to the estimated 76,000 unigenes from the simulation. For avocado, the observed number of unigenes and percent singletons (234,000 and 90%) is considerably larger than predicted for a normalized library sequenced to this depth. We do not know if this unexpected large number of unigenes in avocado is due to a larger underlying transcriptome size, sequence error causing false misassembly [25], or some other unknown factor. For each of these comparisons, we applied the distributions of read lengths, sequence start sites, and transcript abundance frequencies previously observed from Arabidopsis. Therefore, although we have not actually fine-tuned these specific outcomes with the true (and unknown) transcript distribution profiles for each of species and tissue, the observed outcomes are very close to the model predictions. This is particularly true when considering the above uncertainties associated with de novo assembly, differences in tissue sources, and technical variation that may be expected from run to run.

### Analysis of gene expression by NG sequencing

NG sequencing is potentially a direct and cost-effective way to obtain genome scale expression information from organisms that lack a genome sequence and comprehensive microarray platform. Digital expression data obtained by direct sequencing is not dependent on gene models, comprehensive genome data, or understanding of alternative splice forms. The half plate of GS20 sequencing in *Arabidopsis *showed a moderate correlation (r = 0.67, r^2 ^= 0.444, p < 0.0001) between the number of reads and microarray expression values generated from the same tissue and ecotype. Solexa and SOLiD have the potential to increase this correlation, since with millions of sequence reads per experiment, they will ultimately have a large dynamic range similar to traditional microarray experiments. Correct mapping to specific genes may be problematic for short Solexa or SOLiD reads (Figure 5B), when no genome sequence is available. Enriching for 3' UTRs, however, should improve assignment accuracy and increase efficiency of massively parallel sequencing for assessing gene expression levels [38,39]. In research on an organism without a sequenced genome, the value-added expression evidence could be very important in the early stages of developing its transcriptome, an advantage for GS20 or FLX NG technologies. Even in organisms that have comprehensive microarrays, the probe designs are usually dependent on and built with information from early genome assemblies. For example, the current Arabidopsis Affymetrix ATh1 array [40,41] contains probes for approximately 22,000 genes, approximately 5000–6000 genes fewer than the current annotation.

### NG sequencing can be scaled to suit different project goals

NG sequencing technologies are a highly flexible set of platforms that can be used alone or in combination to best suit the research at hand. Small-scale experiments (e.g., 1/16 – 1 plate) provide a wealth of information including the tagging of many or most expressed genes, microsatellite markers, full-length sequencing of highly expressed genes, and modest expression level information. Since there can be multiple lanes in NG plates (up to 16), and multiple bar-coded libraries can be sequenced on a single plate, researchers might fully sequence a small number of highly expressed genes with very little cost or time investment. Low-copy, highly expressed genes might be quite useful for phylogenetic analysis or markers for population level studies. Even small-scale experiments will tag a large fraction of genes in a transcriptome. These tags can be used for building microarray probes [23] and enhancing microarray design. Small studies might sequence the highly expressed genes from many different tissues in the same sequencing runs without the need for bar-coding. Small experiments might also be sufficient to provide rich information for genome annotations in pre-draft and early draft forms. For example, a single run plate will tag nearly every transcribed gene and help identify UTR and intron/exon boundaries. However, experiments on this scale sample only a small fraction of the actual transcriptome and the assembly is often in many small pieces. Moderate transcriptome studies (e.g., 2–5 plates) have the potential to sequence more than 50% of the transcriptome. They will provide small annotation datasets, identify new genes in an organism, further extend genic regions, and help with alternative splicing, especially in sequenced genomes. With deeper sequencing (e.g. 6–20 plates), researchers attain a level of transcriptome that has never been possible before due to the higher cost of earlier technologies. Not only will these studies sequence more than 90% of the transcriptome, the coverage per gene will approach traditional sequencing. This should allow researchers to use these genes to identify pathways, determine tissue-specific expression for lowly expressed genes, and will be critical for genome annotation.

## Conclusion

NG technologies are revolutionizing EST sequencing and applications that revolve around gene expression. Another important consideration in NG transcriptome sequencing is the efficiency and flexibility in library construction. NG library construction costs less, takes less time, and does not produce physical clones that must be stored. If the goal is sequencing full-length genes, non-normalized libraries will yield a larger number of full-length sequences in small sequencing experiments compared to normalized libraries. As sequencing quantities increase, this relationship reverses, and normalized libraries will capture more full-length cDNA sequences. There is also a trade-off in the cost of normalization versus the cost of sequencing. We have shown the feasibility of using a simulation approach to quantitatively evaluate the different platforms and the various combinations of platforms. Currently, the low per-base cost of Solexa sequencing suggests that it may be the most efficient method of transcriptome characterization for sequenced genomes (e.g. Figure 3), but in the absence of a reference genome, the problem of de novo assembly of the short Solexa reads has not yet been resolved. Under these circumstances, a blend of Solexa and GS-FLX sequencing may be optimal (Figure 4).

This is the first simulation study to address some of the technology-specific characteristics found in several NG sequencing technologies. Our approach focuses on the critical questions of data production and coverage, which differ dramatically between methods and experimental scales. By extrapolating the results of the GS20 simulations, we are able to predict outcomes with various NG methods and combinations. ESTcalc allows a variety of assumptions to be explored that will be relevant to different experimental designs with current and future transcriptome sequencing technologies. Although ESTcalc and the underlying simulations do not currently incorporate explicit models of sequencing and assembly errors, the results provide a null hypothesis of predicted outcomes with theoretically perfect sequence data and no assembly error. Deviations from these values in real transcriptome datasets and assemblies will reflect the magnitude of these errors and potential contamination of transcriptome libraries with genomic DNA. These factors will tend to inflate the number of singleton reads relative to the predicted numbers without such errors; their evaluation will aid in sequence cleaning and assembly experiments. A next step will be to develop realistic models of error in sequencing and assembly, and to provide tools to allow any sets of assumptions about read length and cost to be examined. Future studies should be able to build upon this first simulation study, while accounting for additional issues in transcriptome sequencing and assembly experiments.

## Methods

### RNA preparation

*Arabidopsis thaliana *(cv: Landsberg) plants used in this study were grown in a culture chamber at 23 temp and 40% humidity with 18 hours light/6 hours dark. RNA isolation from *Arabidopsis *plants was performed with the RNA Aqueous-Midi kit (Ambion, Inc; catalog: #1911) following the manufacturer's recommendations with modifications as previously reported [27]. California poppy total RNA was prepared from pre-meiotic flower buds using TRIzol reagent (Invitrogen) according to the manufacturer's recommendations. Stages of flower development were defined as described previously [42]. RNA quality and quantity were checked using a Bioanalyzer (Agilent, inc). *Persea americana *pre-meiotic flower buds (Stages 6–7), in which all floral organs are present but stamens and carpels are immature [43], were collected from a tree cultivated on the Gainesville campus of the University of Florida (Kim 1135; voucher deposited at FLAS). Total RNA was isolated using a combination of the CTAB DNA extraction protocol [44] and the RNeasy Plant Mini kit (Qiagen) as previously described for basal angiosperms [45]. RNA integrity was verified with a Bioanalyzer (Agilent Inc.).

### mRNA purification and 454 library construction for *Arabidopsis thaliana *and *Eschscholzia californica*

Messenger RNA was extracted from total RNA using Poly(A)Purist™ mRNA Purification Kit (Ambion, Inc., catalog # 1916) according to the manufacturer's recommendation. mRNA quality was checked with a Bioanalyzer (Agilent, Inc). cDNAs were prepared using the ZAP-cDNA^® ^Synthesis Kit (Stratagene) according to manufacturer's instructions, except that 2 micrograms of mRNA was used rather than the recommended 5 ug. For *Arabidopsis*, two cDNAs were prepared, the first by oligo-dT priming and the second using the random hexamer primers provided in the kit. For California poppy, only random hexamer priming was used to prepare ds cDNA. 454 libraries were constructed from the cDNAs and sequenced using the approach described by [8]. One half plate, one, and two plates were sequenced from Arabidopsis, Persea, and California poppy, respectively, using the 454-G20 sequencer according to manufacturers protocols (Roche, Inc).

### Normalized cDNA library construction in *Persea americana*

Messenger RNA was isolated from 250 ug total RNA using the Poly(A)Purist™ mRNA Purification Kit (Ambion, Inc., catalog # 1916) according to the manufacturer's protocol. Approximately 1 ug high quality mRNA, verified through Bioanalyzer as above, was used to construct a normalized cDNA library using the Trimmer-Direct Kit (Evrogen), which combines a modification of SMART cDNA preparation [46] with DSN-normalization technology [47]. Specifically, first strand cDNA was reverse transcribed using a 3' adapter (CDS-3M; Evrogen) that anneals to poly(A) RNA tails and a second adaptor, BD SMART™ Oligo IV (Clontech), that anneals to the 5' dC tails created by MMLV reverse transcriptase, and serves as an extended template for the first strand synthesis. Double-stranded cDNA was synthesized and simultaneously amplified with the BD SMART™ 5' PCR Primer (Clontech) that anneals to both adaptors through 13 PCR cycles of 95°C for 7 sec; 66°C for 30 sec; 72°C for 6 min on a BioRad thermocycler. Approximately 1.2 ug of double stranded cDNA was purified with the Wizard PCR Purification Kit (Promega) followed by ethanol precipitation, and normalized according to the Trimmer-Direct protocol. Normalized cDNA was subjected to two rounds of single primer PCR amplifications exploiting the complementarity of the cDNA ends (primer sites) to suppress short fragment amplification [48] and enrich the cDNA pool with full length transcripts. The first PCR amplification was conducted for 18 cycles of 95°C for 7 sec; 65°C for 20 sec; 72°C for 6 min. First amplification products with efficient normalization were diluted 10-fold and subjected to 12 PCR cycles of 95°C for 7 sec; 64°C for 20 sec; 72°C for 4 min. Approximately 20 ug of normalized amplified cDNA were thus obtained.

### Sequence analysis

All of the sff files have been deposited into the Short Read Archive at NCBI with the accession SRA008180.19. 454 reads for all species were assembled using the 454 Newbler Assembler [8]. Using the program seqclean http://compbio.dfci.harvard.edu/tgi/software/, we vector trimmed and quality trimmed both the original read files and the contig files generated by the Newbler assembler. We parsed the 454 ReadStatus.txt file to determine the singleton reads, which did not assemble with any other reads. For each library, we used then the contig and singleton files to generate a unigene file. Finally, we calculated the number of sequences, the mean length of all sequences, and the total MB for each of the 4 file types (read, contig, singleton, and unigene). We also performed an additional assembly step using cap3 with 95% identity and 30 bp overlap (default for all other parameters).

Genome mappings for *Arabidopsis *were determined using the best BLASTn [49] match of each individual read versus the TAIR7 genome annotation. We wrote Perl scripts to parse the TAIR xml files containing chromosome locations for all genes in the current annotation. We assigned the following categories to each read: Exon, intron, intron/exon, extended UTR, overlapping genes, intergenic, or no hit. All reads mapping to known gene locations were also mapped to the TAIR cDNA dataset. We calculated the start and stop positions for all reads on each cDNA and determined the reads/gene distribution based on all *Arabidopsis *genes tagged.

In order to evaluate the sequences from the three libraries without a sequenced genome, we used BLASTx and BLASTn searches against the TAIR protein and cDNA datasets, respectively. We parsed the BLAST output to determine the location and e-value to the best Arabidopsis gene. For each best hit, we also determined the length of the protein and/or cDNA, as well as the annotation of the gene. We used the length of the read, the start and stop locations on the gene, and the length of the best hit to calculate each Arabidopsis gene's percent coverage. Gene coverage is defined as the number of bases or amino acids covered by at least 1 read divided by the length of the gene. We estimated the gene coverage using both relaxed and strict definitions. For the relaxed definition, we considered all reads or contigs mapped to a gene, thus allowing for a non-contiguous definition of the gene's coverage. In the strict case, we only considered the longest unigene or read mapped to an individual gene. Please see Additional file 1 for detailed blast results of each dataset used in this study.

### Simulation studies

Using a half plate of GS20 sequences, we developed an approach to simulate additional rounds of sequencing *in silico *as follows. We mapped 118,485 reads to 15,276 genes in the TAIR cDNA dataset using the similarity search program BLASTn. We selected the best high scoring pair (HSP) for each read and its corresponding cDNA. Since there are multiple versions of loci for 3,156 genes, we used the version for each gene with the largest number of reads mapped to its sequence. Using this approach, we created the following distributions: Read length, read count, and cDNA start site distributions. The read length distribution is comprised of all read lengths sampled. The read count distribution contains the number of reads mapped to each gene. For example, the distribution contained 3149, 2238, and 1672 genes with 1, 2, and 3 reads/gene respectively. The most highly expressed gene (AT1G67090, see Table 3) had 586 reads, or about 0.5% of total reads.

We then estimated 3,007 zero class genes by providing the reads/gene profile to the program ESTstat [25]. Zero class genes are defined as genes that have not been sampled (sequenced) but are presumed to be present in the library, but typically expressed at low levels. Failure to account for these genes would bias many of the simulation estimates. We then randomly sampled the zero class genes from the remaining genes not originally sampled. The addition of the zero class genes to the 15,276 transcribed genes totalled 18,283 genes. For the simulation, we used the number of reads per gene distribution to randomly select reads from particular genes based on the number of times they were sequenced. For example, genes that were sequenced only once had a much lower chance of being selected for a plate of simulated reads than genes represented by hundreds or thousands of reads. Finally, we collected all the start sites for each read against its corresponding cDNA to generate a start site distribution. Based on previous work [24,25], we assumed that start sites are dependent on gene length. Based on the quartiles of the Arabidopsis cDNA lengths, we grouped the start sites into four groups: 1) 1–1000 bp, 2) 1001–1500 bp, 3) 1501–2000 bp, and 4) greater than 2000 bp. Using the gene length, we created relative start site distributions dependent on gene length.

The four libraries used in this study were all sequenced using a GS20 machine. In order to compare the GS20 technology to other NG technologies, we also simulated FLX, Solexa, and traditional capillary sequences. For FLX, we used all of the same distributions for the GS20 simulation, except the read length distribution. Since the average read length in our GS20 runs was approximately 100 bp, and the FLX has been reported to generate 250 bp reads, we multiplied the read length of each randomly chosen read by 2.5 for the FLX simulations. In the Solexa simulations, we used a random start site distribution with a 25 bp read length average. We used the same gene expression distribution as the GS20 for all technologies.

To simulate traditional capillary sequences, we downloaded 48,130 ESTs from four different *Arabidopsis *libraries: flower buds, green siliques, roots, and above ground organs 2–6 week old. We partitioned the ESTs into two groups, 5' and 3' based on the annotations located in the fasta header. We then randomly selected 5,000 ESTs from both groups, mapped the transcripts to the *Arabidopsis *cDNA dataset using BLASTn, and generated start site distribution based on cDNA length. We used a 750 bp read length, except when the randomly chosen gene or the length of the gene minus the randomly chosen start site was less than 750 bp. In these cases, we used the gene length or the distance from the start site to the end of the gene as the read length.

In order to compare all technologies, we used conservative estimates (as of late 2008, not including variable costs of library or sample preparation) of the amount (MB) and price ($) of sequencing with each technology. For GS20, an average plate costs $6000/plate and the plate generates 25 MB of data ($240/MB). Since NG machines can be partitioned into smaller segments, we simulated 1/16, 1/8, 1/4, 1/2, 1, 2, 4, and 10 plates for all three technologies. For the GS20, this came to 1.56, 3.12, 6.25, 12.5, 25, 50, 100, 250, and 500 MB increments. For FLX, the cost was calculated using $9000/plate and 100 MB of data ($90/MB), with 6.25, 12.5, 25, 50, 100, 400, 1000, and 2000 MB increments. For Solexa, we used $4000/plate and 1 GB of data ($4/MB) with 50, 100, 200, 500, 1000, 2000, 4000, 10000, and 20000 MB increments. Finally, we converted the cost of traditional capillary sequencing, which is normally calculated per EST (read), by using the conventional $1/EST with 750 bp length ($1330/MB). This included 1.56, 3.12, 6.25, 12.5, 25, 50, 100, and 200 MB increments.

To examine the effects of normalization in next generation transcriptome sequencing, we simulated normalized sequencing for each of the above technologies. We assumed perfect normalization, and changed the gene expression distribution to be equal for all genes. Therefore, of the 18,261 genes estimated to be in the library, each gene has the exact same probability of being chosen as every other gene in the dataset. Although normalization can be technically difficult and requires more labor to generate, we excluded these costs and assumed that there were no additional library costs with normalization sequencing.

To compare the expected simulation results for each technology and combinations of technologies, we calculated the following parameters: percent transcriptome, the number of genes tagged, the total number of unigenes (contigs plus singletons), the mean length of the longest unigene per gene, and the number of genes covered by reads of at least 90% of the length of the gene and in only one unigene. Please see Additional file 2 for detailed simulation results used in this study.

## Authors' contributions

PKW carried out the data analysis and simulation study. ASC, AB, HL, LL, LT, YH generated the data for all GS20 experiments, including the tissue extraction, library construction and optimization at Penn State (SCS, JEC, and CWD labs) and at University of Florida (DES and PSS lab, and the ICBR lab under the direction of William Farmerie). PKW, JLM, HM, JEC, SCS, DES, PSS, NA, CWD conceived of the study, and participated in its design. PKW, JLM, ASC, AB, DES, PSS, NA, CWD coordinated and helped to draft the manuscript. PKW, EW, CWD designed and developed ESTcalc. All authors read and approved the final manuscript.

## Supplementary Material

Additional file 1 **Unigene blast results**. This file contains the detailed blast results of all unigenes. There are 5 worksheets that contain the blast information for each of the GS20 unigene builds, *Arabidopsis *('ath')*, Eschscholzia *('eca – random', 'eca – oligo-dT', and 'eca – combined'), and *Persea *('pam – normalized'). Each worksheet has the following columns: unigene name (query), query unigene length (qlen), number of reads (reads), coverage of best blast result (cov), the database of the best hit (db), the best hit gene name (hit), the length of the hit (hlen), the evalue, bit score, and description of the best hit (desc). See materials and methods for description of the different similarity searches used in these analyses. Click here for file

Additional file 2 **Detailed simulation summary report**. This file contains the detailed simulation results used to generate many of the figures in this article. Simulation results illustrating predicted outcomes for different transcriptome sequencing technologies and combinations of sequencing technologies with a complex library expressing ca. 18,000 genes. The first six fields (tech1, mb1, tech2, mb2, tech3, mb3) contain the technology or combinations of technologies for each simulation along with the amount (mb) of sequencing. For each simulated run, we calculated 16 different parameters: price – the estimated price (per MB) using the following prices for each technology EST5 ($1330), GS20 ($240), GSFLX ($90), and SOL ($4); len -percent of transcriptome sequenced with at least one read and not necessarily in one contiguous sequence; reads – the number of reads in the simulation, sin – percent of reads that are singleton sequences; uni – number of unigenes obtained; puni – number of unigenes divided by the number of estimated genes as a percentage, ulen – mean unigene length (bp)' lulen – mean unigene length (bp) using only the longest unigene per gene; genes – the estimated number of genes tagged; pgenes – the estimated number of genes tagged as a percentage; c90 – the number of genes with more than 90% coverage; c90s – the number of genes with more than 90% coverage and in one contiguous sequence (s = strict); p90s – the percentage of estimated genes with 90% coverage and in 1 contiguous sequence; c100 – the number of genes with 100% coverage; c100s – the number of genes with 100% coverage and in one contiguous sequence (s = strict); p100s – the percentage of estimated genes with 100% coverage and in one contiguous sequence. Click here for file
